# Annelid Distal-less/Dlx duplications reveal varied post-duplication fates

**DOI:** 10.1186/1471-2148-11-241

**Published:** 2011-08-16

**Authors:** Carmel McDougall, Natalia Korchagina, Jonathan L Tobin, David EK Ferrier

**Affiliations:** 1 The Scottish Oceans Institute, University of St Andrews, East Sands, St Andrews KY16 8LB, UK; 2 Department of Zoology, University of Oxford, South Parks Road, Oxford OX1 3PS, UK; 3 School of Biological Sciences, University of Queensland, Brisbane, Queensland 4072, Australia

## Abstract

**Background:**

Dlx (Distal-less) genes have various developmental roles and are widespread throughout the animal kingdom, usually occurring as single copy genes in non-chordates and as multiple copies in most chordate genomes. While the genomic arrangement and function of these genes is well known in vertebrates and arthropods, information about Dlx genes in other organisms is scarce. We investigate the presence of Dlx genes in several annelid species and examine Dlx gene expression in the polychaete *Pomatoceros lamarckii*.

**Results:**

Two Dlx genes are present in *P. lamarckii, Capitella teleta *and *Helobdella robusta*. The *C. teleta *Dlx genes are closely linked in an inverted tail-to-tail orientation, reminiscent of the arrangement of vertebrate Dlx pairs, and gene conversion appears to have had a role in their evolution. The *H. robusta *Dlx genes, however, are not on the same genomic scaffold and display divergent sequences, while, if the *P. lamarckii *genes are linked in a tail-to-tail orientation they are a minimum of 41 kilobases apart and show no sign of gene conversion. No expression in *P. lamarckii *appendage development has been observed, which conflicts with the supposed conserved role of these genes in animal appendage development. These Dlx duplications do not appear to be annelid-wide, as the polychaete *Platynereis dumerilii *likely possesses only one Dlx gene.

**Conclusions:**

On the basis of the currently accepted annelid phylogeny, we hypothesise that one Dlx duplication occurred in the annelid lineage after the divergence of *P. dumerilii *from the other lineages and these duplicates then had varied evolutionary fates in different species. We also propose that the ancestral role of Dlx genes is not related to appendage development.

## Background

Dlx genes are homeobox genes that were first discovered in *Drosophila melanogaster *[1] and are best known for their role in appendage development in a wide range of taxa [2-4]. This role is one of several, however, as Dlx genes also have roles in nervous system development and early embryogenesis [2,5-10]. Dlx genes are widespread throughout Metazoa and are found in early branching lineages such as cnidarians [11-13] and placozoans [14]. It appears, therefore, that the Dlx gene evolved early in metazoan evolution, before the divergence of protostomes and deuterostomes, but probably after the divergence of sponges, which most likely lack a Dlx gene [15].

Only one Dlx gene has been discovered in the genome thus far in protostomes, echinoderms, and cephalochordates [6,10,16]. Therefore, it is likely that a single copy of the gene is the ancestral state for bilaterians. Mice and humans have three pairs of Dlx genes, which exist in a tail-to-tail arrangement linked to a Hox cluster (the HoxC cluster has no linked Dlx genes; [2]). It is thought that the ancestral chordate Dlx gene was linked to the Hox cluster, underwent a gene-specific duplication and inversion, and the Dlx gene pair was then duplicated during the whole genome duplications that occurred in the vertebrate lineage [2,17,18]. In support of this hypothesis, it appears that Dlx2, Dlx3 and Dlx5 form one paralogous group and that Dlx1, Dlx4 and Dlx6 form another [17,19]. The urochordate *Ciona intestinalis *possesses three Dlx genes, two of which are arranged in a tail-to-tail orientation. All three of the genes are closely linked to *CiHox13 *and *CiHox12 *(the Hox cluster is dispersed in *C. intestinalis*, and *CiHox13 *and *CiHox12 *exist as a bigene cluster; [20]). The divergent nature of the *C. intestinalis *Dlx sequences has made the deduction of clear gene orthologies difficult, but it is thought that the paired ascidian Dlx genes are a result of the same duplication that led to the paired arrangement of Dlx genes in the vertebrates [20]. The cephalochordate amphioxus possesses only one Dlx gene, which is linked to the Hox cluster [21] and is thought to represent the pre-Dlx duplication state. To date, there are no documented cases of Dlx gene duplications outside the chordates.

The bulk of our knowledge about Dlx gene expression, function, and genomic location is from vertebrates, where Dlx has a large range of roles, including the control of limb formation, differentiation of neuronal subsets and various novel functions relating to the neural crest, such as the development of craniofacial structures (reviewed in [2]). Much less is understood about Dlx genes in invertebrates, but the information that is available comes primarily from *D. melanogaster*, and partly from other arthropods and the nematode *Caenorhabditis elegans *[5,6,22-24], all of which belong to only one of the two protostome super-phyla, the Ecdysozoa. Our understanding of Dlx in the second protostome super-phylum, the Lophotrochozoa, is more rudimentary. A cross-reactive Dlx antibody [25] has been used in both molluscs and annelids, and distinct staining patterns are consistent with these organisms possessing a Dlx gene [3,9]. Fragments of Dlx have been cloned from two molluscs [26], and a Dlx gene has been isolated in the annelids *P. dumerilii *[27] and *Neanthes arenaceodentata *[28]. Interestingly, the three Dlx expression studies performed in annelids show somewhat different expression patterns; in *Chaetopterus variopedatus *the Dlx antibody recognises regions in the parapodial rudiments as well as in the neurogenic ectoderm [3], in *N. arenaceodentata in-situ *hybridisation shows *NvDll *expression in the proximal part of appendages and in the brain [28], whereas in *P. dumerilii in-situ *hybridisation indicates that *PduDlx *(referred to in the original paper as *PduDlx1*) is expressed in broad regions in the lateral ectoderm which is interpreted to be at the border of neurogenic and non-neurogenic ectoderm [27]. Each of these studies examine restricted developmental stages, making comparisons between the organisms difficult. Thus, the function of Dlx in annelids is poorly understood, as is the extent of its variation between species. In addition, there is no published information about the genomic organisation of Dlx genes in any lophotrochozoan species.

Here we undertake a survey of Dlx genes in the polychaete annelids *P. lamarckii *and *P. dumerilii*, and identify Dlx genes in the genome assemblies of *C. teleta *and *H. robusta*. All of these species, with the exception of *P. dumerilii*, possess two Dlx genes. In *C. teleta*, the two Dlx genes exhibit a vertebrate-like tail-tail gene pair arrangement and show evidence of gene conversion, whereas there is no evidence for the close linkage of *P. lamarckii *or *H. robusta *Dlx genes, which correlates with a lack of evidence for gene conversion in these species. We propose that a duplication of an ancestral Dlx gene took place early in annelid evolution, after the divergence of the *P. dumerilii *lineage from the other annelid lineages, and the subsequent divergence of the fates of these duplicated genes. The *P. lamarckii *genes are expressed in presumptive neural cells, but are not detected during appendage development. The duplication of Dlx genes and their apparent absence from appendage development mean that further characterisation of invertebrate Dlx genes is needed, and that evolutionary scenarios based on the assumption of single Dlx genes in protostomes and a near universal role in appendage development need to be re-assessed.

## Results

### *P. lamarckii *Dlx genes

**Figure 1:**
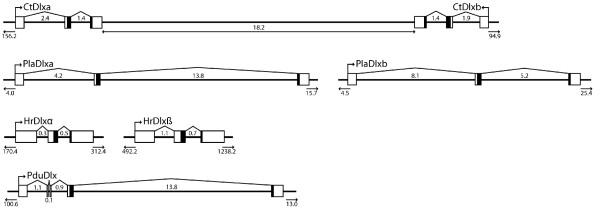
**Genomic arrangement of annelid Dlx genes**. Exons are indicated by boxes, introns are indicated by lines adjoining these. Distances are given in kb, the homeodomain is shaded in black. The distance to the end of the scaffold or contig is indicated at the ends of each schematic.

**Figure 2:**
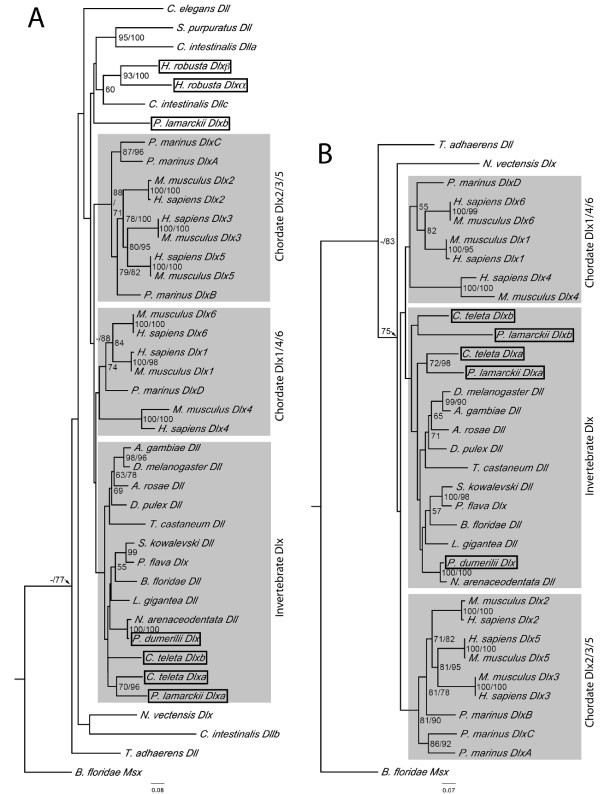
**Neighbour-joining analysis of the phylogenetic relationships of annelid Dlx genes**. JTT method of substitution with 1000 bootstrap replicates. Percentage of bootstrap support is shown when over 50%, followed by Bayesian posterior probability values where these are over 70%. The trees are rooted with *BfMsx*, branch lengths are to scale (scale = substitutions/site). Sequences from representative species include *Anopheles gambiae *(arthropod), *Athalia rosae *(arthropod), *Branchiostoma floridae *(cephalochordate), *Caenorhabditis elegans *(nematode), *Ciona intestinalis *(ascidian), *Drosophila melanogaster *(arthropod), *Daphnia pulex *(crustacean), *Homo sapiens *(vertebrate), *Lottia gigantea *(mollusc), *Mus musculus *(vertebrate), *Neanthes *arenaceodentata (annelid), *Nematostella vectensis *(cnidarian), *Ptychodera flava *(hemichordate), *Petromyzon marinus *(chordate), *Saccoglossus *kowalevski (hemichordate), *Strongylocentrotus purpuratus *(echinoderm), *Trichoplax adhaerens *(placozoan), *Tribolium castaneum *(arthropod). Sequences that are the subject of this study are boxed. A. Analysis including highly divergent sequences. There is some support for the homology of *C. teleta *and *P. lamarckii *Dlxa sequences but the relationships of the other annelid Dlx sequences are unknown. *H. robusta Dlxα and Dlβ *group together with other sequences with longer branch lengths, possibly as a result of long branch attraction. B. Analysis excluding highly divergent sequences. As well as the grouping of *C. teleta *and *P. lamarckii *Dlxa genes, the *Dlxb *genes of these two species now also show an affinity, but without strong support values.

Shotgun sequencing of Dlx positive phage clones and subsequent RACE on identified homeodomain sequences revealed two distinct genes with homology to Dlx. *PlaDlxa *[Genbank: accession numbers JN175271 and JN175273] encodes a 380 amino acid protein, whereas *PlaDlxb *[Genbank: accession numbers JN175272 and JN175274] encodes a 396 amino acid protein. Each gene encodes a 60 amino acid homeodomain and is comprised of three exons, with the second intron between amino acids 44 and 45 of the homeodomain (Figure 1). Phylogenetic analysis clearly places both genes within the Dlx clade (Figure 2). Genomic walking by library screening focussed on determining whether these genes are linked in a tail-to-tail arrangement and demonstrates that, if so, the two genes are a minimum of 41 kb apart (Figure 1). Southern hybridisation and direct sequencing of other Dlx positive phage clones failed to identify any additional Dlx sequences.

**Figure 3:**
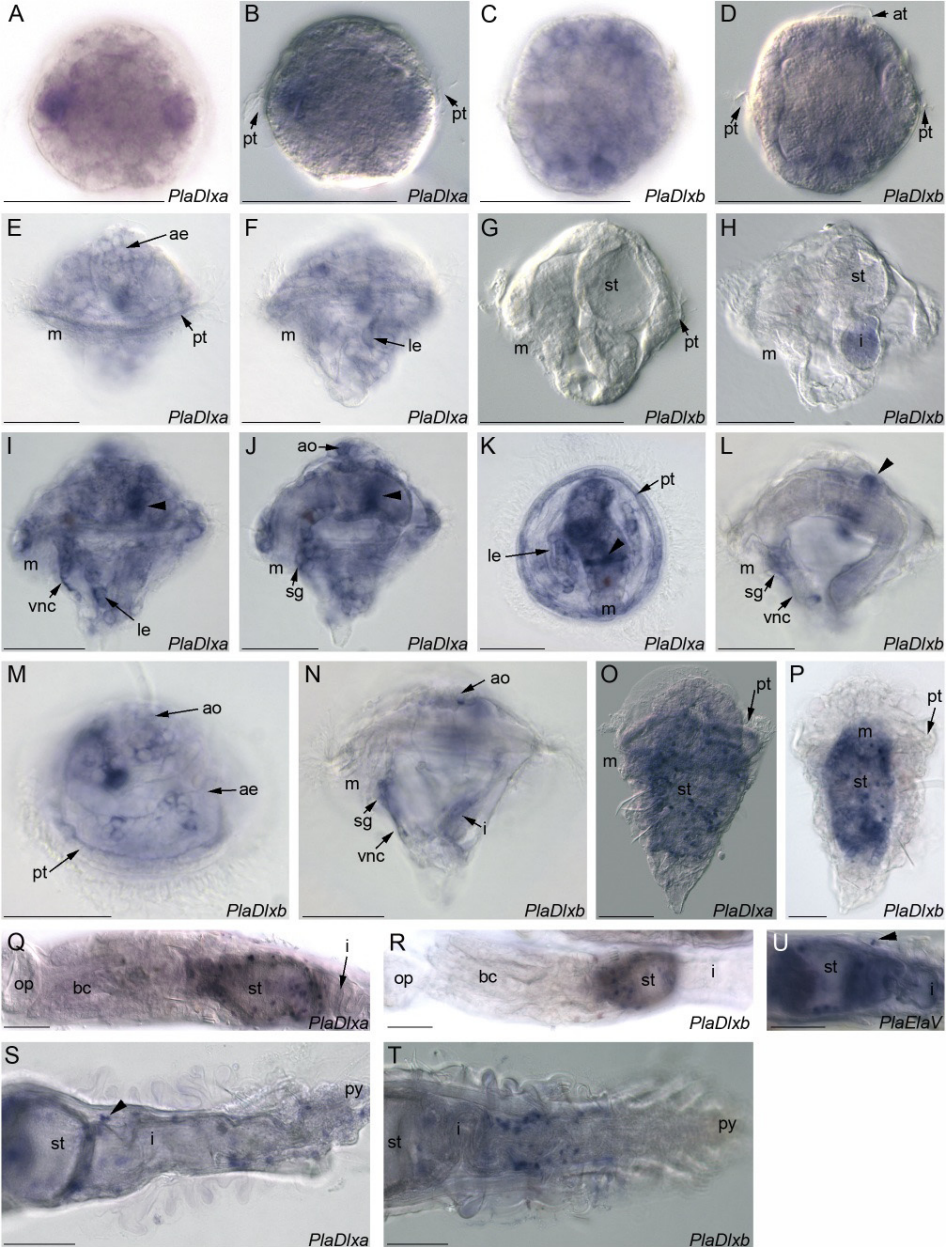
***PlaDlxa *and *PlaDlxb *expression during *P. lamarckii *development**. Scale bars = 50 μm. The mouth (m) is indicated as a reference point. A. Early embryo, brightfield. *PlaDlxa *expression is higher in some cells, which seem to vary between individuals. B. Same embryo as A, DIC. The prototroch is apparent, therefore these Dlx positive cells are mid-lateral. C. Early embryo, brightfield. *PlaDlxb *is expressed in multiple cells. D. Same embryo as C, DIC. Prototroch and apical tuft are clear, therefore *PlaDlxb *expression is in both episphere and hyposphere. E. Early trochophore, lateral view focused on episphere. *PlaDlxa *is expressed in apical ectoderm and prototroch. F. Same larva as E, hyposphere in focus. *PlaDlxa *is also expressed in lateral ectoderm. G. Early trochophore, lateral view. No *PlaDlxb *expression is detected. H. Early trochophore, more developed than G. No *PlaDlxb *expression is detected. The small patch of colouration in the intestinal lumen is attributable to background staining. I. Complete trochophore, lateral view. *PlaDlxa *is expressed in ventral nerve cords, lateral ectoderm, and a band around the stomach (arrowhead). J. Same larva as I, different focal plane. *PlaDlxa *is expressed in the suboesophageal ganglion and apical organ. The broad band around the stomach is still visible (arrowhead). K. Complete trochophore, ventral view. *PlaDlxa *expression in the prototroch is clear, as is the band around the stomach (arrowhead) and the lateral ectoderm. L. Complete trochophore, lateral view. *PlaDlxb *is expressed in the suboesophageal ganglion, ventral nerve cords, and a ring around the stomach (arrowhead). M. Complete trochophore, apical-lateral view. *PlaDlxb *is expressed in apical ectoderm, apical organ and prototroch. N. Complete trochophore, lateral view. *PlaDlxb *is expressed in the apical organ, suboesophageal ganglion, ventral nerve cords and intestine. O. Metatrochophore, lateral view. *PlaDlxa *has a punctate pattern throughout the animal. P. Metatrochophore, ventral view. *PlaDlxb *is primarily expressed in cells in the stomach area. Q. Young juvenile, ventral view. *PlaDlxa *is expressed in cells associated with the stomach. R. Young juvenile, dorsal view. *PlaDlxb *is expressed in cells associated with the stomach. S. Posterior of older juvenile, ventral view. *PlaDlxa *expression is in cells in the stomach, intestinal region, in individual cells in the pygidium and between intestinal folds (arrowhead). T. Posterior of older juvenile, ventral view. *PlaDlxb *expressing cells in the intestinal region. U. Older juvenile, *PlaElaV *(a neural marker) expression shown for comparison. *PlaElaV *is expressed in cells associated with the stomach, in some cells in the intestinal region, and in some cells in the parapodia (arrowhead). Apical ectoderm (ae), apical organ (ao), apical tuft (at), branchial crown (bc), intestine (i), lateral ectoderm (le), operculum (op), prototroch (pt), pygidium (py), stomach (st), suboesophageal ganglion (sg), ventral nerve cords (vnc).

*In-situ *hybridisation of *PlaDlxa *and *PlaDlxb *showed that both genes are expressed in isolated cells in the early embryo, and in the apical ectoderm, prototroch, lateral ectoderm, ventral nerve cords, apical organ, sub-oesophageal ganglion and in a band around the stomach in the trochophore larva (Figure 3A-N). At the metatrochophore stage, expression of both genes becomes punctate and is primarily located in discrete cells in the vicinity of the stomach (Figure 3O,P). In early juvenile (post-metamorphosis) animals, *PlaDlxa *and *PlaDlxb *continue to be expressed in cells around the stomach; in older animals the majority of expression can be found in discrete cells near the intestine (Figure 3Q-T). No expression is observed in the parapodia at any stage. In general, the expression of the two genes is very similar, although there may be down-regulation of the expression of *PlaDlxb *at the early trochophore stage whilst *PlaDlxa *is still detectable (Figure 3 G,H). In all cases where *Dlx *is detected, the precise pattern of cells expressing *Dlx *genes is variable between individuals, suggesting expression of *Dlx *is transient and dynamic. Control hybridisations with two non-overlapping probes for each gene gave consistent, comparable results (data not shown) and the same protocol with different genes gave clearly distinct staining patterns, as well as hybridisations with no probe producing no staining at all (Additional File 1).

The *ElaV *gene is a commonly used neuronal differentiation marker. We isolated a 670 bp region of this gene [Genbank: accession number JN175270], phylogenetic analyses show that it is closely related to *P. dumerilii *ElaV (Additional File 2). *PlaElaV *was also expressed in a punctate pattern in the stomach of juvenile *P. lamarckii *animals, reminiscent of the pattern seen for the *P. lamarckii Dlx *genes (Figure 3U).

### *C. teleta *Dlx genes

**Figure 4:**
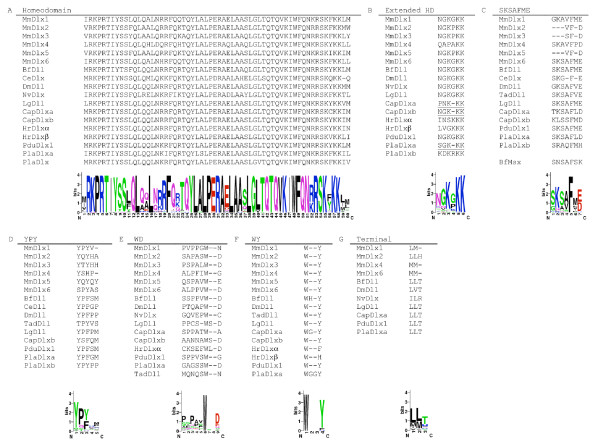
**Conserved Dlx motifs**. Alignment of representative sequences, beneath each alignment is a sequence logo corresponding to the alignment of all Dlx sequences used to generate the phylogenetic tree in Fig 1. The overall height of each column corresponds to the overall sequence conservation at that position, and the height of each letter corresponds to the overall frequency of that amino acid. Sequence logos were created using Weblogo [93]. A. Homeodomain alignment of representative Dlx genes. B. Conserved residues upstream of the homeodomain. Note the shared gap in *CtDlxa, CtDlxb *and *PlaDlxa *(underlined) C. The SKSAFME motif, located close to the N-terminus of the protein. A similar motif can be found in the same location in *BfMsx*. D. The YPY motif. E and F. Two conserved tryptophan residues are found downstream of the homeodomain, the first is generally followed by an aspartic acid, and the second by a tyrosine. G. Hydrophobic terminal motif, at or near the C-terminal of the protein. Species abbreviations are as follows: *Branchiostoma floridae *(Bf), *Caenorhabditis elegans *(Ce), *Ciona intestinalis *(Ci), *Drosophila *melanogaster (Dm), *Lottia gigantea *(Lg), *Mus musculus *(Mm), *Nematostella vectensis *(Nv), *Trichoplax adhaerens *(Tad).

BLAST searches of the *C. teleta *trace files on NCBI and Genscan predictions based on genomic sequence of both the assembled genome on the JGI website and of a genomic contig generated manually from trace files resulted in the identification of two putative Dlx genes. One of these genes shares some conserved sequence motifs with *PlaDlxa *(see below and Figure 4) and also groups with it in the phylogenetic trees (see Figure 2), therefore we have designated it *CtDlxa*. The second gene appears more divergent and has hence been named *CtDlxb*. While the majority of the sequence of *CtDlxa *and *CtDlxb *is quite divergent from each other, sequence similarity within the homeodomains is very high. Specifically, the nucleotide sequence of the homeoboxes are identical 5' of the homeobox intron (and for the first 12 nucleotides of this intron), and there are only three nucleotide differences at the 3' end, giving an overall similarity of 98.4% within this region.

While several different gene models are put forward for each gene, the Fgenesh ab initio models (*CtDlxa*: fgenesh1_pg.C_scaffold_237000015, *CtDlxb*: fgenesh1_pg.C_scaffold_237000013) predict an intron-exon structure typical for Dlx genes (three exons, with the second intron located between amino acids 44 and 45 of the homeodomain, Figure 1). As there are no EST sequences corresponding to either *CtDlxa *or *CtDlxb *these predictions cannot currently be confirmed. *CtDlxa *and *CtDlxb *are located adjacent to each other in a tail-to-tail orientation on scaffold 237 of the whole genome assembly. In this assembly, the intergenic distance is 18,352 bp, however there are some gaps within this region. In order to confirm the gene arrangement and intergenic distance, we completed our own assembly of *C. teleta *genomic trace files spanning these genes. This *de novo *assembly confirmed the tail-to-tail orientation of *CtDlxa *and *CtDlxb*, and puts the intergenic distance at 18,202 bp. It must be noted that some prediction methods identify a gene within this intergenic region (fgenesh1_pg.C_scaffold_237000014). However, this is only predicted by a few of the methods, it has no homology to any known sequence, and is not confirmed by EST's. We therefore consider it to be a false prediction.

### *H. robusta *Dlx genes

As with *C. teleta*, BLAST searches of the *H. robusta *trace files on NCBI and Genscan predictions based on genomic sequence of the assembled genome on the JGI website resulted in the identification of two putative Dlx genes. Both sequences group with other Dlx genes in phylogenetic trees (Figure 2A). Outside the homeodomain, there is very little conservation between either *H. robusta *Dlx gene and any other Dlx gene; the genes have therefore been arbitrarily designated *HrDlxα *and *HrDlβ*. None of the gene models in the JGI assembly appear to predict the correct coding sequence for either *HrDlxα *or *HrDlβ *(the sequences are either truncated or missing part of the homeobox, but see fgenesh4_pg.C_scaffold_92000036 for the best prediction of *HrDlxα*, and fgenesh4_pg.C_scaffold_41000062 for the best prediction of *HrDlβ*), and there are no corresponding EST sequences for this region. The coding sequences were therefore predicted by running approximately 10 kb of surrounding sequence through the GENSCAN program, which indicates that both genes are comprised of three exons with the second intron located between amino acids 44 and 45 of the homeodomain (Figure 1). In each case the sequences are much longer than the other annelid Dlx sequences (*HrDlxα *and *HrDlβ *encode predicted proteins of 860 and 871 amino acids, respectively), and contain many poly-amino acid tracts, predominately polyglutamine.

*HrDlxα *can be found on scaffold 92, which is 486 kb in length. Fgenesh ab initio models predict a homeobox-containing neighbour of this gene, a likely Pknox family member. This gene is 23 kb away from *HrDlxα *and there are two other predicted genes in this intergenic distance. This is the only other homeobox gene on this scaffold. *HrDlβ *can be found on scaffold 41, which is 1.73 Mb in length. There are no other homeobox genes on this scaffold. From the genome assembly it is clear that the two *H. robusta *Dlx genes are not closely linked; if they are on the same chromosome the minimum distance between the two genes is approximately 659 kb.

### *P. dumerilii *Dlx genes

A Dlx gene (*PduDlx *- Genbank AM114774) has previously been identified from *P. dumerilii*, it consists of five exons, including two microexons (which differs from the previously mentioned Dlx genes, Figure 1). Library screening was performed to determine whether a second Dlx gene is present in the *P. dumerilii *genome. All BAC and phage clones that produced a positive signal possessed *PduDlx*, no additional Dlx sequences were obtained.

One *PduDlx *positive BAC clone has previously been completely sequenced [29], Genbank CT030672. This sequence was run through the online Genscan program [30] in order to predict open reading frames (ORFs). 10 ORFs were predicted by Genscan, none of these contained a homeobox sequence. Two of the predicted open reading frames are similar to each other (22% identity, 49% positives) and both show high similarity to the 'ORF2' region of a zebrafish LINE element (accession AB211149; [31], Additional Files 3, 4 and 5).

### Conserved Dlx motifs

In addition to the homeodomain and a number of amino acids adjacent to it (the 'extended homeodomain'), there are several other regions of conservation within the Dlx protein (Figure 4). The most notable of these is located close to the N-terminal of the protein and has been designated the 'SKSAFME' motif. This domain is present in most Dlx genes except for those in *Nematostella vectensis, C. intestinalis, Strongylocentrotus purpuratus *and *H. robusta *(the *Petromyzon marinus *sequences appear to be incomplete at the 5' end of the gene and were therefore excluded). The mammalian Dlx 2/3/5 clade possesses some residues with identity to this motif but lack the full sequence. A similar sequence is also found at the N-terminal of the *B. floridae *Msx gene which was included in this study as an outgroup. Other conserved motifs include the 'YPY' motif, which is N-terminal to the homeodomain, two regions of conservation each surrounding a tryptophan residue C-terminal to the homeodomain, and a hydrophobic domain at or near the C-terminus of the protein.

### Evolutionary relationships between annelid Dlx sequences

In order to understand the relationship between the various Dlx genes and duplicates discovered above, a neighbour joining tree was created using an alignment of the homeodomain and its flanking sequences as well as some of the conserved motifs mentioned above from a range of taxa (for alignment see Additional File 6). Bayesian analysis was also performed. The resulting tree (Figure 2A) recovers the expected topology for mammalian Dlx genes, grouping Dlx 2/3/5 and Dlx 1/4/6 (although the latter group had weak bootstrap support). Arthropod Dlx genes formed a clade (with the exclusion of *Tribolium castaneum Dll *which had weak support) and the two hemichordate Dlx genes (*Saccoglossus kowalevskii Dll *and *Ptychodera flava Dlx*) were grouped together with high support. Annelid Dlx genes were generally not grouped together, although *CtDlxa *and *PlaDlxa *formed a well-supported clade. Several of the more divergent sequences (such as the *C. intestinalis *and *H. robusta *Dlx genes) were grouped together, suggestive of long branch attraction. The analysis was therefore repeated without these genes, which resulted in very little change to the topology of the tree, except that *PlaDlxb *now grouped with *CtDlxb *with low support (Figure 2B).

### Gene conversion in *C. teleta*

**Figure 5:**
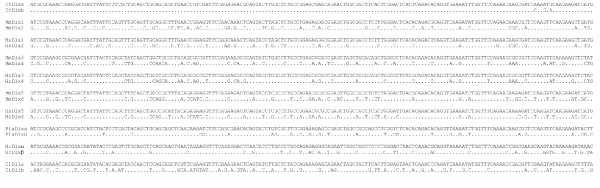
**Sequence similarity at the nucleotide level in paired Dlx genes**. Pairwise alignments of the homeobox from Dlx genes that are arranged as inverted pairs, as well as *H. robusta *and *P. lamarckii *Dlx genes. Conserved nucleotides are indicated by a dot. Nucleotide similarity is strikingly higher in the *C. teleta *Dlx genes than in any other gene pair.

From the Dlx sequence alignments it became apparent that the two *C. teleta *Dlx genes demonstrated extremely high sequence similarity in the homeodomain region. This similarity was also observed at the nucleotide level. In order to examine whether this was a general feature of Dlx genes in taxa where two Dlx genes exist in an inverted pair, alignments of the nucleotide sequences of the homeoboxes were performed for each pair in each species (Figure 5). From this alignment, it is evident that the sequence identity seen between the two *C. teleta *genes does not exist in any of the other gene pairs investigated.

In order to test the significance of the amount of identity seen in the *C. teleta *Dlx genes, nucleotide alignments were run through the program Geneconv [32], see Additional File 7 for results. This program identified a fragment of 150 bp (corresponding to the 5' part of the homeodomain and part of the intronic sequence) which is highly likely to be undergoing gene conversion in *CtDlxa *and *CtDlxb*. A second region corresponding to the 3' part of the homeodomain was also identified, however this had a tract length of only 31 bp. Ten other significant tracts were identified in both the *C. teleta *and *P. lamarckii *Dlx genes, however these were very short, ranging from 21-10 bp in length. The biological significance of these is uncertain given the level of sequence conservation normally seen in homeoboxes. The GC content of the tracts identified by Geneconv did not differ from the remainder of the sequence.

## Discussion

### Functions of conserved Dlx motifs

The identification of several Dlx genes from annelids and comparisons with other taxa allowed the identification of several highly conserved domains within these sequences. These regions of amino acid conservation in Dlx genes from distantly related taxa are presumably functionally important. The SKSAFME domain is particularly well conserved and is situated close to the N-terminal of the protein. In many other homeodomain containing genes, a domain in this position is responsible for transcriptional regulation of downstream targets [33-35]. This domain has been identified individually in many classes of homeodomain genes and therefore is known by several names, including the Hep motif [35], octapeptide [36], TN domain [37], eh1 homology region [33,38], SNAG domain [39] and NK decapeptide [40]. A similar domain (*HNF-3*) is found in unrelated Forkhead genes [41]. Apart from their shared location within the gene, some sequence similarity is evident when many different domains are aligned (see Additional File 8, adapted from [42], consensus sequences from [33,35-38,41]). The SKSAFME domain found in Dlx genes has previously been called the 'Hep' domain [14,43], and shows some sequence similarity to these regions (particularly those found in Vent and Msx genes) in the alignment. It is therefore possible that the SKSAFME domain in Dlx genes functions to control transcription of downstream target genes.

As well as the Hep motif, many homeobox genes also encode a conserved hexapeptide motif located N-terminal of the homeodomain which encodes a central tryptophan residue [38,44]. This motif, which is also called the PID domain, is involved in binding PBX protein cofactors, increasing the specificity of DNA binding by the homeodomain [45,46]. No conserved motifs containing a tryptophan can be found upstream of the homeodomain in Dlx genes, however the two conserved tryptophan residues 3' of the homeodomain (Figure 4) may fulfil this cofactor binding role.

### Annelid Dlx duplicates - one duplication?

To date, duplicated Dlx genes have only been found in chordate lineages, therefore the discovery of multiple Dlx genes in several annelid lineages is surprising. In particular, the similarity in arrangement of *C. teleta *Dlx genes with those found in chordates (i.e., in an inverted tail-to-tail pair) is intriguing and poses the question of whether the annelid and chordate Dlx pairs arose as the result of an ancient Dlx duplication which has been followed by the loss of one gene in multiple other lineages (for example, in ecdysozoans, ambulacrarians and cephalochordates). Also there is a question as to whether there is some kind of selective advantage or constraint associated with having tandem duplicates linked in this way, or whether the arrangement has occurred by chance alone.

The phylogenetic analysis presented here does not present any evidence to suggest that the chordate and annelid Dlx genes arose from a common gene duplication, but there is limited resolution due to the relatively short sequence aligned and few phylogenetically informative residues, a problem commonly encountered with Dlx phylogenetic trees [28,47]. In any case, there is no evidence of close linkages of annelid Dlx genes with Hox genes, unlike the situation seen in chordates; the Pknox gene found near *HrDlxα *is distantly related to the Hox genes and unlikely to be significant, and while *PduDlx *is on the same chromosome as the Hox cluster in *P. dumerilii*, it is quite distant from it (Hui et al., submitted). While *P. dumerilii *and, presumably, the closely related *N. arenaceodentata*, appear to possess only one Dlx gene, all other annelids examined in this study possess two. These annelid duplicates could be the result of 1) independent duplications, 2) of a pre-annelid duplication followed by gene loss in *P. dumerilii*, or 3) by a duplication that occurred after the divergence of the *P. dumerilii *lineage from that of the other annelids studied. We favour the third scenario, given the grouping of *PlaDlxa *and *CtDlxa *(and, to a lesser extent, *PlaDlxb *and *CtDlxb*) in the phylogenetic trees and the similarities seen within the sequences, i.e. the possession of one relatively prototypical Dlx gene which possesses the common motifs, and one more divergent Dlx. In addition, the *C. teleta *and *P. lamarckii *Dlx genes share some unusual changes, such as a deletion in the extended homeodomain motif, and a shared aspartic acid at the end of their SKSAFME motif (see Figure 4). We therefore tentatively conclude that the presence of two Dlx genes in these species is a consequence of a single duplication in the ancestor of *C. teleta *and *P. lamarckii*, rather than the slightly less well supported possibility that independent duplications in each lineage were followed by the divergence of one gene and the stasis of the other. The *H. robusta *Dlx genes are quite divergent from other annelid Dlx genes, therefore it is unclear whether the Dlx genes of *H. robusta *arose in an independent duplication or whether they may also be descended from a single duplication that gave rise to the *C. teleta *and *P. lamarckii *Dlx genes.

**Figure 6:**
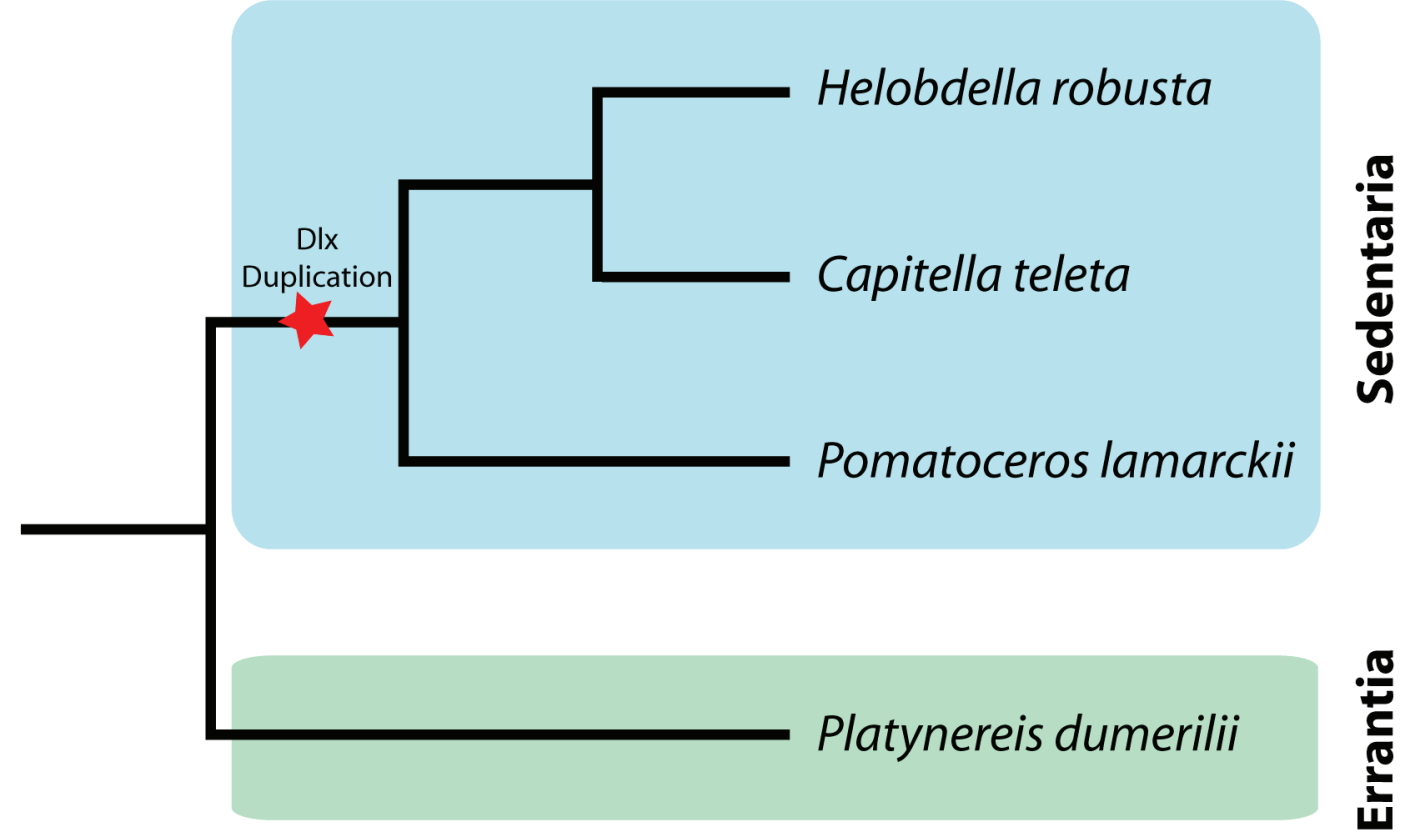
**Hypothesized location of the Dlx gene duplication in an annelid phylogeny**. The annelids depicted are restricted to those examined for Dlx content in this work. The topology of the phylogeny is based upon [48].

If the *C. teleta, P. lamarckii*, and possibly the *H. robusta *Dlx genes arose in a single duplication, is the sole Dlx gene in *P. dumerilii *a result of gene loss or divergence in the nereid lineage prior to the duplication? A recent paper has examined relationships between annelid families, and has demonstrated that, in general, annelids belong to one of two major clades, the Errantia (which includes *P. dumerilii*), or the Sedentaria (which includes *C. teleta, P. lamarckii *and *H. robusta*) [48]. Therefore, the most parsimonious hypothesis using the available data is that *P. dumerilii *diverged prior to a duplication in the lineage leading to *C. teleta, H. robusta *and possibly *P. lamarckii *(Figure 6). Further information regarding the Dlx gene complement of other Errantia species is required to confirm this proposal.

If the chordate and annelid duplicates are indeed independent, then the duplicated Dlx genes have converged on the same tail-to-tail genomic arrangement in at least two independent events. There are several other examples of homeobox genes being organised in this convergently transcribed manner, such as the engrailed and invected genes in hexapods [49], and the iroquois genes in multiple organisms [50,51]; these show both tail-to-tail and head-to-head organisation. Therefore, it appears that having duplicates arranged in this way occurs often and may well have some kind of selective advantage or constraint. Sharing of enhancer elements has been proposed for the engrailed and iroquois examples mentioned above, and, in vertebrates, enhancer sharing has been demonstrated for the Dlx1/2 and Dlx5/6 gene pairs [52-55]. Such enhancer sharing can result in similar expression patterns, and may lead to selective advantage by allowing more precise transcriptional control of similar transcripts that act in a combinatorial manner, such as the Hox genes [56-59]. Therefore, the paired *C. teleta *and chordate Dlx genes may be the result of parallel evolution of a favourable gene arrangement. It is interesting to note that the *P. lamarckii *Dlx genes have very similar expression patterns, it will be interesting to discover whether this is due to linkage and enhancer sharing of the two genes once more genomic data is available.

### Duplicated annelid Dlx genes have divergent fates

*CtDlxa *and *CtDlxb *are closely linked in a tail-to-tail organisation and are predicted to encode two quite divergent proteins. Despite this, there is 100% similarity between the nucleotide sequences of the *C. teleta *Dlx genes in the 5' (pre-intron) part of the homeobox, and in the 5' part of the intra-homeobox intron. There is also high similarity in the 3' part of the homeobox. The striking nucleotide identity between the two copies is higher than expected even if the sequences were constrained due to selection, especially as there is also conservation in silent sites. In addition, if selection were responsible for the maintenance of sequence a higher degree of similarity would be expected between *C. teleta *Dlx genes and those from other, related species. Figure 5 shows that this is clearly not the case. An alternative explanation is that the similarity is due to the genes being a product of a recent duplication, however this is unlikely as the remainder of the gene exhibits a high level of divergence.

This unexpectedly high level of conservation and the closely linked and inverted physical arrangement of the two genes (which is reminiscent of many other examples of unexpected nucleotide conservation in duplicated genes in the literature) is consistent with a proximity-based gene conversion mechanism. Gene conversion is the 'non-reciprocal transfer of information from one DNA duplex to another' [60], and concerted evolution by gene conversion has been documented in several *D. melanogaster *gene duplicates, such as Hsp70 and Hsp82 [61,62], *α*-amylase [63,64], and trypsin [65], and also in putative antimicrobial proteins in *C. elegans *[66] and within the extensive palindromic sequences on human and chimp Y chromosomes [67]. Each of these examples documents gene conversion between genes that are closely linked in an inverted orientation. Examples of gene conversion in non-inverted duplicates also exist, for example, Nv1 neurotoxin genes in *N. vectensis *[68] and rRNA genes in *D. melanogaster *[69], as do examples of conversion of genes on different chromosomes [70].

Gene conversion could therefore explain the high nucleotide identity between the two *C. teleta *Dlx genes. The program Geneconv is a statistical test for gene conversion, and it identifies 4 tracts that are likely to have undergone gene conversion in the *C. teleta *Dlx sequences and 8 tracts in the *P. lamarckii *Dlx sequences. However, the majority of these tracts are extremely short. It is likely that a minimum length of sequence homology is required for gene conversion to take place, it has been reported that at least 50 bp of homology are required ( [71] and references therein) and other studies have used a minimum tract length of 100 bp to search for genes undergoing gene conversion [72]. Despite this, very short (< 12 bp) gene conversion tract lengths have been reported for yeast [73]. In addition, the identification of gene conversion based on sequence similarity is complicated by the fact that the homeobox is a highly conserved sequence, therefore tracts of homology could occur by chance. Given that *CtDlxb *and *Homo sapiens Dlx1 *exhibit a tract of perfect homology of 25 bp, it is clear that this degree of similarity is indeed possible and can occur by chance alone. It is therefore likely that the phenomenon of gene conversion is restricted only to the 5' part of the homeobox and some of the adjoining intron of *CtDlxa *and *CtDlxb*, and possibly also to the 3' part of the homeodomain, i.e, the region of the gene which is most highly conserved across taxa. This 'mosaic' pattern of gene conversion within a gene is well documented [72], and gene conversion in homeobox genes has been described before, in the hexapod engrailed and invected genes [49]. While duplicated Dlx genes are seen elsewhere in the animal kingdom, the *C. teleta *Dlx genes are the only known example where gene conversion seems to be taking place (see Figure 5). The mammalian genes, despite also being arranged in a tail-to-tail orientation and having a shorter intergenic distance [53,55] show a much higher level of sequence divergence in the homeobox. This might be explained by higher rates of evolution in these genes, which would allow them to 'escape' gene conversion [66,74]. Mammalian Dlx4 genes have been shown to have elevated sequence divergence (in comparison to other mammalian Dlx genes), possibly due to reduced selection pressure because of the redundancy that exists within mammalian Dlx genes [75]. This redundancy may also allow elevated evolution rates in the other mammalian Dlx genes.

Within the annelids, *H. robusta *and *P. lamarckii *also have duplicated Dlx genes which do not appear to be subject to gene conversion. The *H. robusta *genes are not closely linked, are much longer than Dlx genes found in other species, and lack most of the conserved motifs found in other Dlx genes. It therefore appears that the *H. robusta *Dlx genes have been subject to much higher rates of evolution than those of other annelid species, and that any selective pressure or constraint to keep the Dlx duplicates together has been overcome in this species.

While the genomic arrangement of *P. lamarckii *Dlx genes is unknown, they are at least 41 kb apart if they are tail to tail, or 8.5 kb apart if they are head to head. Therefore, if they are linked in a tail to tail orientation they are not as closely linked as the *C. teleta *Dlx gene pair. There is no evidence of gene conversion between *PlaDlxa *and *PlaDlxb*. While *PlaDlxa *shows similar branch lengths to *CtDlxa*, the branch lengths of *PlaDlxb *are significantly longer, indicating a higher rate of evolution of this particular gene. The similar expression patterns of *PlaDlxa *and *PlaDlxb *indicate that the two genes may be co-expressed, possibly pointing to shared cis-regulatory sequences if the two genes are indeed linked.

Regardless of whether they are the result of a single or multiple gene duplications, the paired Dlx genes of these annelid species are not behaving in a similar manner post-duplication. In the current post-genomic era, large-scale studies are being conducted in order to understand the dynamics of gene duplication and the effects of these events on the evolution of the organisms involved. General trends have been difficult to identify, and it appears that the chances of a newly duplicated gene being retained in the genome is a largely neutral process [76]. From the example of duplicated Dlx genes in annelids, we can once again observe that there is no general pattern evident in their sequence evolution that explains the behaviour of duplicated genes.

### The role of Dlx genes in annelid development

This study has found that Dlx genes are unlikely to be playing a major role in appendage formation in *P. lamarckii*. Throughout development, *PlaDlxa *and *PlaDlxb *are expressed in what is interpreted to be neural tissue, but the expression is dynamic and turned off in various structures (such as the ventral nerve cords) after their formation. We therefore hypothesise that *PlaDlxa *and *PlaDlxb *are involved in the differentiation of the nervous system. In support of this, the *P. lamarckii *homologue of ElaV, a neural differentiation marker [27,77,78] is expressed in a punctate and dynamic pattern in the juvenile stomach, which is very similar to Dlx.

While Dlx is expressed in the parapodia of another polychaete, *C. variopedatus *[3], it is not clear that this expression is indicative of a role in the process of appendage formation or if it is associated with the development of sensory or neural structures. Indeed, in *N. arenaceodentata*, Dlx expression observed at the base of the parapodia is interpreted as being associated with the parapodial ganglia [28]. In all four annelids studied to date, Dlx expression is observed in what is assumed to be developing neural tissue. Dlx expression is also observed in early embryogenesis in *P. lamarckii*; where this has been demonstrated in other organisms it has been implicated in the control of cellular movements during gastrulation [8-10,79]. Despite the expression of *P. lamarckii *Dlx genes in widely conserved expression domains, the absence of an appendage formation role is surprising. There is a possibility that a third *P. lamarckii *Dlx gene exists and is involved in appendage formation (however this is unlikely given the thoroughness of the screening) or that the lack of Dlx expression in the appendages in *P. lamarckii *represents a taxon specific loss.

It is important to note that while Dlx is expressed in the appendages of many organisms, in some cases 'limb' Dlx is more likely to be playing a role in limb-associated neural structures [5]. In some cases it has been shown that Dlx is not an absolute requirement for appendage outgrowth. Dlx knockouts have been performed in spiders; in injected embryos appendages do form but they lack the most distal region [23]. In fact, some arthropod appendages do not exhibit any Dlx expression at all [80]. The vertebrate condition is complicated by the redundant nature of the multiple Dlx genes, and single gene mutants have no visible defects in limb formation. Combinatorial Dlx mutants exhibit malformations of the distal limb (reviewed in [2]), but the limb itself still forms. Therefore, the 'conserved' appendage function of Dlx genes relates to its role in the development of the distal appendage. Perhaps, then, the distal appendage has been lost in the evolution of annelid parapodia, or gained independently in arthropods and vertebrates. There are clear examples of the Dlx gene being co-opted into the formation of novel appendage-like structures, such as echinoderm tube feet and ascidian siphons [3,81], so a gain of function in both arthropods and vertebrates is certainly possible.

Dlx is associated with the nervous system in a multitude of taxa. Therefore, this is a much more likely ancestral role for the gene than is limb formation. This is supported by the very early origin of the gene in metazoan diversification, i.e, prior to the radiation of bilaterians, the ancestor of which may have lacked limbs altogether (see [5] for discussion]. However, in some cases Dlx expression has been observed very early in embryogenesis [[8,9], this study], therefore we suggest that the ancestral role of Dlx could also be in a specific type of morphogenetic process that is utilised in numerous ways throughout animal development. A similar hypothesis has been put forward by Irvine and colleagues [20]. One potential morphological process is evagination, as Dlx expression is seen during early embryonic stages (gastrulation), which may use similar cellular movements to the evaginations required during appendage formation [20]. Another potential morphogenetic process that Dlx may be involved in is cell adhesion. In *C. elegans*, RNAi knockdown of Dlx (*ceh-43*) causes the loss of cells though a hole in the hypodermis and an eventual rupture of the animal [6], therefore a role of Dlx in cell adhesion was proposed. Interestingly, cell adhesion can be mediated by neurons, which can act as guidance cues for cellular movements [6,82], providing a potential link for Dlx in both morphogenetic processes and in the nervous system. Elements of either, or both, of these processes may then have been co-opted into the process of appendage formation in several taxa.

## Conclusions

We have presented here the first examples of duplicated Dlx genes outside the chordates. We propose that a duplication of the Dlx gene occurred within the annelid lineage, after the split of *P. dumerilli *from the lineage leading to *H. robusta, C. teleta *and *P. lamarckii*. The two *C. teleta *Dlx genes are closely linked and have been subject to gene conversion, the two *H. robusta *Dlx genes are not closely linked and exhibit divergent gene sequences, and the *P. lamarckii *genes do not show gene conversion, but have very similar expression patterns. Therefore, in these three cases, the duplicated Dlx genes have had very different post-duplication fates.

## Methods

### Animal sources and library construction

Adult *P. lamarckii *were collected and spawned as described [83]. Genomic DNA was extracted from sperm and a phage library was created by Lofstrand Labs, Maryland, USA using the LambdaFIX II (*Xho1*) vector, and amplified. Genomic DNA was partially digested with *Sau2A1*, filled in and cloned. The average insert size of the library was calculated to be 16-17 kb. XL1-Blue MRA(P2) cells (Agilent) were then transfected and plated to give approximately 4× genome coverage for library screening by hybridisation.

Sperm from a single male *P. dumerilii *worm was prepared in agarose plugs. DNA was then extracted from the plugs before being sent to Loftstrand Labs for library construction as outlined above. The average insert size was also calculated to be 16-17 kb. This library was plated to give approximately 5× genome coverage for screening by hybridisation.

Details of the *P. dumerilii *BAC genomic library can be found in [29].

### Library screening

A 5' fragment (811 bp) of *P. dumerilii *Dlx (accession AM114774.1) was generated from a cDNA clone using specific primers (PdDLL5' - 5' GGG ATT ACA GCC TGA GAC and PdDLL3' - 5' TTT ACC TGA GTT TGG GTG), and a labelled probe was synthesised using the PCR DIG labelling mix (Roche) following the manufacturer's instructions. The *P. lamarckii *genomic phage library was then screened for Dlx using standard methods [84] with a hybridisation temperature of 37°C and two post-hybridisation washes in 2× SSC, 0.1% SDS for 15 minutes at room temperature (RT), followed by two washes in 0.5× SSC, 0.1% SDS for 15 minutes at 55°C. Signals were detected using a 1:20,000 dilution of anti-digoxygenin-AP (Roche) and CDP-Star chemiluminescent substrate (Roche) as per the manufacturer's instructions. Two phage plaques producing strong signals and giving different restriction digest patterns were chosen for complete sequencing. Each clone was sonicated and A-overhangs added. They were then ligated into the pGem-T Easy vector and sequenced using T3 and T7 primers. Vector trimming was performed manually and contig assembly was performed using the DNAStar software (low stringency settings, no vector trimming). Gaps in the phage insert sequences were closed by sequencing using specific primers. These sequences were checked for homology to known Dlx genes using BLASTX, and intronic arrangement was predicted using the GENSCAN software http://genes.mit.edu/GENSCAN.html. In order to determine if the two discovered Dlx genes were closely linked, genomic walking was performed by designing probes to the end sequences of the parental phage and screening the library as detailed above, except that hybridisation temperatures were raised to 42°C and post-hybridisation washes were increased to 65°C. Resulting phage sequences were checked for the presence of Dlx genes by PCR, and long range PCR was performed using the Expand Long Template PCR kit (Roche) in order to determine phage length.

The *P. dumerilii *genomic phage and BAC libraries were screened using the same Dlx probe and conditions as outlined above. Positive BAC clones were ordered from BACPAC resources and checked for the presence of *PduDlx *by PCR using the specific primers shown above. The degree of overlap of BAC sequences was determined by end-sequencing using vector primers.

### Manual trace assembly

The *C. teleta *and *H. robusta *trace archives http://www.ncbi.nlm.nih.gov/Traces/trace.cgi were searched for Dlx by using the discontiguous megaBLAST algorithm and the homeodomain and flanking sequences of *PlaDlxa *and *PlaDlxb *as the query sequence. Entire *C. teleta *and *H. robusta *Dlx sequences and their orientations were obtained by 'walking' from the trace files obtained in the above searches. This involved blasting the terminal 200 bp of sequence against the trace archive, downloading overlapping traces and assembling the contigs using the SeqMan assembler (DNAStar suite). Assembly settings were default, with a medium level of end trimming, a minimum match size of 12 and minimum match percentage of 80.

### Rapid Amplification of cDNA Ends

RACE-ready cDNA libraries were constructed from mixed larval *P. lamarckii *total RNA and RACE performed using the BD SMART RACE cDNA Amplification Kit (Clontech) as per the manufacturer's instructions, except that the annealing temperatures for the touchdown PCR were lowered (see brackets after each primer listing for initial annealing temperatures). Primer sequences were as follows:

*PlaDlxa *5': 5' CGACAGGTACTGTGTTCGCTGGAAGATC (70°C)

*PlaDlxa *5' nested: 5' GAGGAGTAGATGGTGCGGGGCTTGCGGA (66°C)

*PlaDlxa *3': 5' GAGAGAGCCAGATGAGCCCACGCCCAAG (70°C)

*PlaDlxa *3' nested: 5' CGGTCTCACACAGACACAGGTNAARATH (60°C)

*PlaDlxb *5': 5' GCATTTGACCTTGGTTCTGCGAGGGAAT (70°C)

*PlaDlxb *5' nested: 5' TTCACCTGAGTCTGTGTGACGCCAAGGC (66°C)

*PlaDlxb *3': 5' AGAATGAACCTGGCATATCCTCCAAGGA (70°C)

*PlaDlxb *3' nested: 5' GCCTTGGCGTCACACAGACTCAGGTGAA (66°C)

### Phylogenetic analysis

Dlx sequences (and the *B. floridae *Msx sequence, which was used as an outgroup) were retrieved from NCBI http://www.ncbi.nlm.nih.gov/. Accession numbers can be found in Additional File 9. Sequences were formatted in BBEdit Lite 6.1 [85] before being aligned using clustalx [86] with reduced gap penalties (for pairwise alignments the gap opening penalty was set to 5 and the gap extension penalty to 0.05; for the multiple alignment the gap opening penalty was set to 5 and the gap extension penalty to 0.1). Alignments were manually edited using Se-Al v2.0 [87]. Two alignments were produced, one with a comprehensive Dlx dataset and another with the most divergent taxa removed in order to reduce the probability of tree topology disruption. Phylogenetic trees were built using the Phylip 3.66 package of programs [88]. A neighbour joining tree was constructed using the JTT matrix with 1000 bootstraps, and a consensus tree produced. Bayesian analysis was performed using MrBayes v3.1.2 [89], with two runs for 2.5 (full dataset) or 1.5 (dataset with divergent taxa removed) million generations (sampled every 100, first 250 trees discarded as burn-in) using the mixed amino acid substitution model and the gamma likelihood model for among-site rate variation. Trees were viewed and edited using FigTree [90].

### Detection of gene conversion

Alignments of genomic Dlx sequences from *C. teleta, P. lamarckii, P. dumerilii *and *H. robusta *(padded by an additional 5000 bp both up and downstream) were performed using the program CHAOS with DIALIGN [91]. The alignment was searched for regions of potential gene conversion between *C. teleta *or *P. lamarckii *sequences using the program GENECONV [32] with default settings except that monomorphic sites were included.

For regions for which gene conversion was deemed to be likely, GC content was determined manually for the third codon positions of exons and for intronic sequence.

### *In-situ *hybridisation

Larvae and juveniles were cultured as described [83] and fixed according to a previously reported protocol [92]. Larvae 36 hpf or older were relaxed by the addition of an equal amount of 7% MgCl_2 _in FSW to the dish contents. Probes were synthesised using DIG RNA labelling mix (Roche) according to the manufacturer's instructions. For each gene probes were designed 3' of the homeodomain, the *PlaDlxa *probe was 491 bp long and synthesised using the primers *PlaDlxa Fwd *(5' CCCTCTAACCCCACAGCCTCCG) and *PlaDlxa Rev *(5' CCGTAGCCACCCCAGCCCCCGT), the *PlaDlxb *probe was 576 bp long and synthesised using *PlaDlxb Fwd *(5' TTCCCTCGCAGAATCAAGGTCA) and *PlaDlxb Rev *(5' CGCCACCATACGGGTAATAACC). The *PlaElaV *sequence was isolated via degenerate touchdown PCR using the primers ElaV-1 (5' CGMTAYGGSTTYGTNAACTA) and ElaV-2 (5' BACDGCBCCRAANGGNCCRAA) with an annealing temperature of 60°C which was decreased by 0.5°C per cycle for 40 cycles. The resulting product was used as a probe and labelled as outlined above.

Prior to hybridisation fixed animals were stepped into cold PBT (1× PBS, 0.1 M Tween-20) with 5 minute washes. Juveniles were decalcified in PBTE (1× PBS, 0.1 M Tween-20, 0.05 M EGTA) for approximately 30 minutes until calcified tube was no longer visible. Samples were then treated with 0.5 μg/ml proteinase K at 37°C for 10 minutes and were post-fixed in 4% paraformaldehyde in 1× PBS for 45 minutes at room temperature. Hybridisation was performed as described in [92], but using a hybridisation temperature of 55°C. Negative controls that lacked probe were performed and showed no staining (see Additional File 1). The reproducibility of the *PlaDlxa *and *PlaDlxb *stainings was confirmed by using two probes from different regions of each gene (data not shown) as well as by comparison to non-neural genes with different patterns of staining and which do not stain the gut (see Additional File 1), thus confirming that the staining around the gut in *PlaDlxa *and *PlaDlxb *experiments is unlikely to be due to probe trapping and instead is likely to reflect staining of visceral nerve cells. Stained specimens were cleared in 60% glycerol and photographed using a Zeiss Axioskop 2 and the Axiovision 4 software or a Zeiss Axioplan 2 and the Openlab software.

## Authors' contributions

CM carried out molecular genetic studies, participated in the design of the study and drafted the manuscript. JT performed the initial Dlx probe synthesis and library screen of the *P. lamarckii *phage library. NK sequenced the initial Dlx positive *P. lamarckii *phage clones and performed the first round of genomic walking in this organism. DEKF conceived of the study, participated in its design and was involved in drafting the manuscript. All authors have read and approved the final manuscript.

## Supplementary Material

Additional file 1 **In-situ hybridisation controls**. Negative in situ controls lacking probe and positive controls with an unrelated gene. Click here for file

Additional file 2 **Phylogenetic position of *PlaElaV***. Neighbour-joining tree showing the relationships between *ElaV *genes from different taxa. Click here for file

Additional file 3 **Consensus sequence of PduL2**. Sequence derived from alignment of sequences found in several different BAC clones. Click here for file

Additional file 4 **The domain organisation of PduL2**. Graphic depicting the endonuclease and reverse transcriptase domains of PduL2. Click here for file

Additional file 5 **Phylogenetic position of PduL2**. Neighbour joining tree showing the relationships between PduL2 and other LINE elements. Click here for file

Additional file 6 **Dlx alignment**. Alignment of homeodomain and other conserved regions of Dlx genes used for phylogenetic analysis. Click here for file

Additional file 7 **Geneconv output**. Tracts of Dlx sequences likely to be undergoing gene conversion as predicted by Geneconv. Click here for file

Additional file 8 **Hep-like domains**. Alignment of N-terminal Hep-like domains of selected homebox and forkhead genes. Click here for file

Additional file 9 **Sequences used in phylogenetic analyses**. Accession numbers and database sources for sequences used in phylogenetic analyses. Click here for file
