# Supplementary material for: Annelid Distal-less/Dlx duplications reveal varied post-duplication fates
Source: BMC Evol Biol. 2011 Aug 16;11:241. doi: 10.1186/1471-2148-11-241 (PMC3199776; doi:10.1186/1471-2148-11-241)
Supplement: Additional file 1 — In-situ hybridisation controls. Negative in situ controls lacking probe and positive controls with an unrelated gene. [file 1471-2148-11-241-S1.PDF]

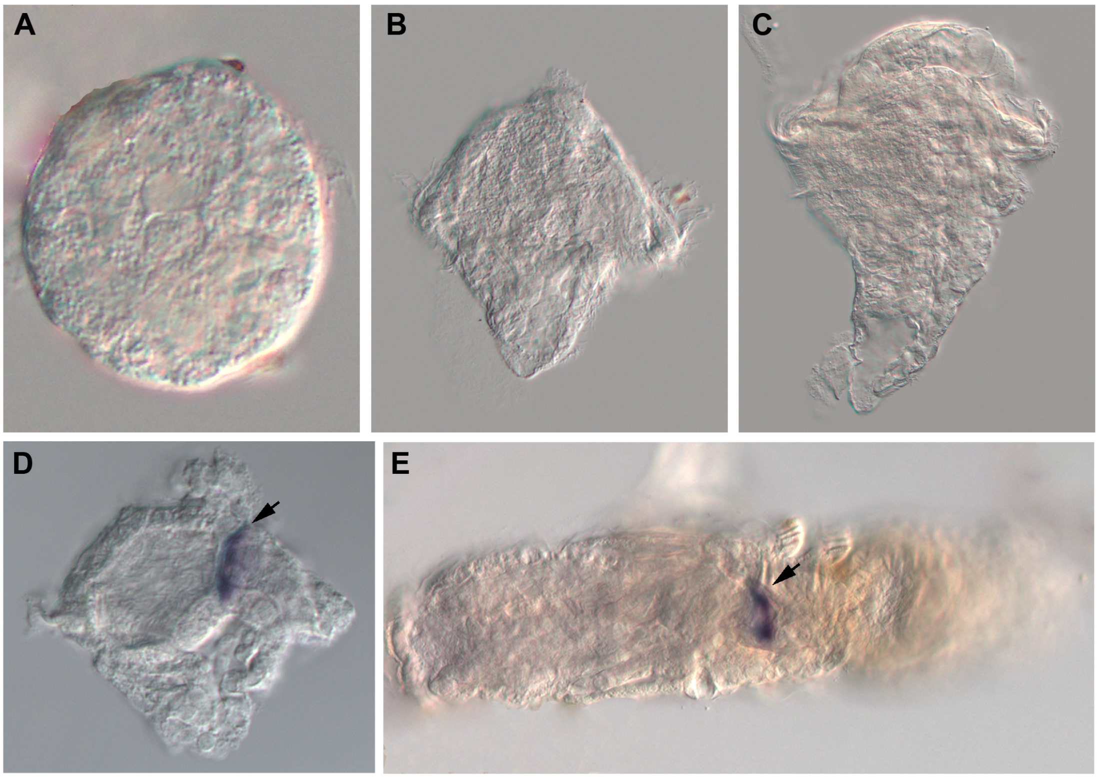

Additional file 1. *In-situ* hybridisation controls.
A, B, C. No probe controls of early embryo, complete trochophore, and metatrochophore, respectively, demonstrating the lack of any staining. D, E. Control probe *in-situ* hybridisation showing a staining pattern distinct from that of *Dlx* or *ElaV*.
