# Supplementary material for: Annelid Distal-less/Dlx duplications reveal varied post-duplication fates
Source: BMC Evol Biol. 2011 Aug 16;11:241. doi: 10.1186/1471-2148-11-241 (PMC3199776; doi:10.1186/1471-2148-11-241)
Supplement: Additional file 2 — Phylogenetic position of PlaElaV. Neighbour-joining tree showing the relationships between ElaV genes from different taxa. [file 1471-2148-11-241-S2.PDF]

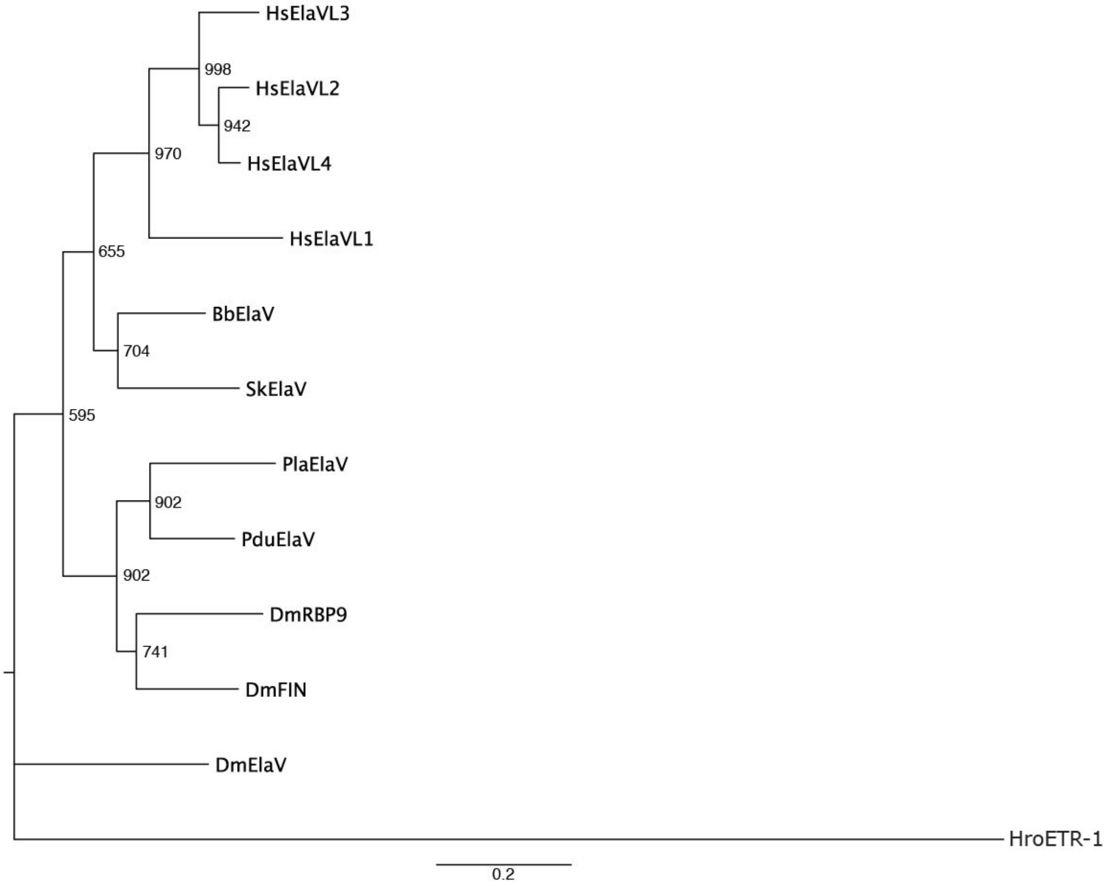

Additional File 2. Phylogenetic position of *PlaElaV*.

PlaElaV was aligned to ElaV family genes from other taxa, *PlaElaV* groups with *PduElav* with high support. All accession numbers are from Genbank. *HsElaVL1,* NP_001410.2 (*Homo sapiens*); *HsElaVL2,* NP_004423.2 (*Homo sapiens*); *HsElaVL3,* NP_001411.2 (*Homo sapiens*); *HsElaVL4*, NP_068771.1 (*Homo sapiens*); *BbElaV,* BAB62225.1 (*Branchiostoma belcheri*); *SkElaV,* AAP79277.1 (*Saccoglossus kowalevskii*); *PduElaV*, ABO93208.1 (*Platynereis dumerilii*); *DmRBP9,* NP_476937.2 (*Drosophila melanogaster*); *DmFIN*, NP_001096965.1 (*Drosophila melanogaster*); *DmElaV*, AAF45517.2 (*Drosophila melanogaster*); *HroETR-1,* BAB40781.1 (*Halocynthia roretzi*). Percentage bootstrap support (1000 replicates) is shown when over 50%. The tree was rooted with *HroETR-1*, branch lengths are to scale.
