# Supplementary material for: Annelid Distal-less/Dlx duplications reveal varied post-duplication fates
Source: BMC Evol Biol. 2011 Aug 16;11:241. doi: 10.1186/1471-2148-11-241 (PMC3199776; doi:10.1186/1471-2148-11-241)
Supplement: Additional file 3 — Consensus sequence of PduL2. Sequence derived from alignment of sequences found in several different BAC clones. [file 1471-2148-11-241-S3.PDF]

```
>PduL2
LSPGTNCKANYSISSKANFGFLIFEIAKKHEKLKLDSDRLGLGLKKLDNKDKPAIPKHKF
QRSIDSQIKLLKNFEDALQDEDKLANAENDGNHLCKNELRILKACKMCAQNAQQKREKTK
NVKDEFDEDDFDFFEFTEKTDDDDDIGEIEELEENFELFFLREGRPGGGGGCIGCIGLKA
IKADQKKRMKFEHFEHMEHLIGKDGKILLVLLIEPPEKNKKKKSEFAEEFQEFTAHLHAQ
HNENNEIGDFGDFMFHMDDADDLKFIKFLNIFDFEDCVQHSTHKDGNDLDLVITHITQEL
IACAVSDLLSDHNCVLFDIAEIKGKKAQKEISFRKTRDINLIEFHKDIEDHLSQKLQCGD
KSEHLNELVALYNSTKQILDKHAPVITKEVTLRKPTPFTNADIKNLKTAKRKAERLWRRT
GLEKDWDDFKEKRNALNEHLNQLKSDDLRSKIESTKGNSKAMFKLLNSSLNRKQELPLPI
HTNEKDLANDFNSFFDEKIKTIRSKLDSCNDETADAEEFAGHKLFQFKALSKAEVKKLIG
NMPVKHCQLDPIPTWLLLECIDAFLPIITEIVNTSLTLGEMPLDLKHALVKPLLKKAGLD
LIKKNYRPVSNLSFLGKIIESAVIKQYMDHLTRNKLDDDKQSAYKQFHSTETLLTKIHND
IMLNMSKGEVTMLVLLDLSAAFDTIDHNILLKRLKNRYGVQDSALNWFKSYISERSQSVC
IHDTTSEKLPLKFGVPQGSKLGPILFNSYIAPVSEVAAQNQVDDQKYADDEQLILSFKPT
FLEQENAVDKMEKCIAEIRKFLHENKLCNNGDKTELLLIGSPHQLNKLQVSSINVDNVEI
KAADHVRNLGVIFDKNMTMEKQVNKMCRNAYFNLRNISISKIRKNKDKEKTKTAVNALVT
PHLDYGNGLLYGSSLINKLKLQVAQNSAVRLIEKLEDHDSVSHHRKHLHWLPIPARIQFK
LMTWTWKAGNAQAPIYILILIQPKPERNLNLRSKAKLLLPEPASCNKNKDEDFAFSAPAP
KLWLPLPEKVRKTKTLEGFKKKLKTYLFFKFYRNIN
```

Additional File 3. Consensus sequence of PduL2.

The ORF identified in the *PduDlx* containing BAC was used in pairwise BLASTp alignments against other *P. dumerilii* BAC sequences deposited in Genbank and also several unpublished BAC sequences. Positive hits were obtained from BAC sequences with the Genbank accession numbers CT030671, CT030672, CT030679, CT030681, and two unpublished BACs. The consensus sequence is displayed above.
