# Supplementary material for: Annelid Distal-less/Dlx duplications reveal varied post-duplication fates
Source: BMC Evol Biol. 2011 Aug 16;11:241. doi: 10.1186/1471-2148-11-241 (PMC3199776; doi:10.1186/1471-2148-11-241)
Supplement: Additional file 4 — The domain organisation of PduL2. Graphic depicting the endonuclease and reverse transcriptase domains of PduL2. [file 1471-2148-11-241-S4.PDF]

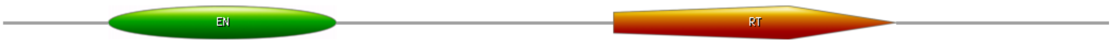

Additional File 4. The domain organisation of PduL2.
The consensus sequence is 1056 amino acids in length and possesses two domains, an endonuclease (EN) domain and a reverse transcriptase (RT) domain.
