# Supplementary material for: Annelid Distal-less/Dlx duplications reveal varied post-duplication fates
Source: BMC Evol Biol. 2011 Aug 16;11:241. doi: 10.1186/1471-2148-11-241 (PMC3199776; doi:10.1186/1471-2148-11-241)
Supplement: Additional file 5 — Phylogenetic position of PduL2. Neighbour joining tree showing the relationships between PduL2 and other LINE elements. [file 1471-2148-11-241-S5.PDF]

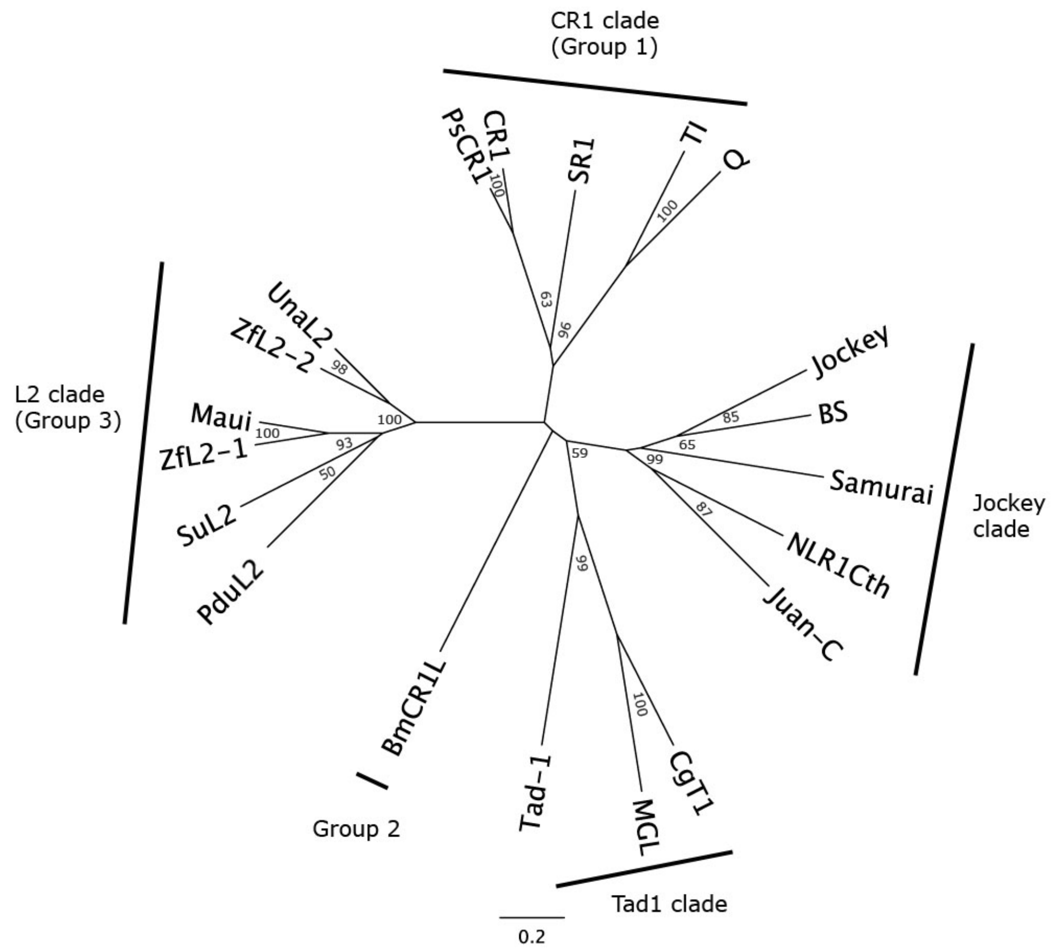

Additional File 5. Phylogenetic position of PduL2.
PduL2 was aligned with other LINE elements. PduL2 is included in the L2 clade with high support. Neighbour-joining, JTT method of substitution. Percentage bootstrap support (1000 replicates) is shown when over 50%. Branch lengths are to scale. Genbank accession numbers are as follows: CR1, U88211; PsCR1, AB005891; SR1, U66331; T1, M93689; Q, U03849; MGL, AF018033; CgT1, L76169; Tad-1, L25662; BS, X77571; Jockey, M22874; NLR1Cth, S59870, Juan-C, M91082; Samurai, AB055391; UnaL2, AB179624; SuL2, AAGJ01206634; Maui, AF086712; BmCR1L, AB159446 ZfL2-1, AB211149; Zfl2-2, AB211150.
