# Supplementary material for: Annelid Distal-less/Dlx duplications reveal varied post-duplication fates
Source: BMC Evol Biol. 2011 Aug 16;11:241. doi: 10.1186/1471-2148-11-241 (PMC3199776; doi:10.1186/1471-2148-11-241)
Supplement: Additional file 6 — Dlx alignment. Alignment of homeodomain and other conserved regions of Dlx genes used for phylogenetic analysis. [file 1471-2148-11-241-S6.PDF]

```
          1         11        21        31        41        51        61        71        81        91        101       111       121       131       141
          |         |         |         |         |         |         |         |         |         |         |         |         |         |         |
MmDlx2    ----VF-DSLYQYHADKEDLE-PEIRINGKPKKVRKPRTIYSSFQLAALQRRFQKTQYLALPERAELAASLGLTQTQVKIWFQNRRSKFKKMWKSGEIPTEQHSAPASWDFLGWYSHLQATAP-LLHPSQTPQPVSAGTIF
HsDlx2    ----VF-DSLYQYQAEKEDLE-PEIRINGKPKKVRKPRTIYSSFQLAALQRRFQKTQYLALPERAELAASLGLTQTQVKIWFQNRRSKFKKMWKSGEIPSEQHSAPASWDFLGWYSHLQATAP-LLHPTQTPQPVSAGTIF
MmDlx5    ----VF-DRRYQYQYEKEVAE-PEVRMNGKPKKVRKPRTIYSSFQLAALQRRFQKTQYLALPERAELAASLGLTQTQVKIWFQNKRSKIKKIMKNGEMPPEHSQSPAVWEYLEWYSHLPPPGS-LQHP-----ALASGTLY
HsDlx5    ----VF-DRRYQYQYEKEVTE-PEVRMNGKPKKVRKPRTIYSSFQLAALQRRFQKTQYLALPERAELAASLGLTQTQVKIWFQNKRSKIKKIMKNGEMPPEHSQSPAVWEYLEWYSHLPPPGS-LQHP-----ALASGTLY
MmDlx3    ----SF-DRKYTYHHVKEEPE-AEVRMNGKPKKVRKPRTIYSSYQLAALQRRFQKAQYLALPERAELAAQLGLTQTQVKIWFQNRRSKFKKLYKNGEVPLEHSPSPALWDYLDWYPHLQQPAT-LHHASP---PPNPGAVY
HsDlx3    ----SF-DRKYTYHHVKEEPE-AEVRMNGKPKKVRKPRTIYSSYQLAALQRRFQKAQYLALPERAELAAQLGLTQTQVKIWFQNRRSKFKKLYKNGEVPLEHSPSPALWDYLDWYPHLQQPAT-LHHASP---PPNPGAVY
PmDlxC    ----VF-DCGYPY-QEKEERE-SGVRMNGKPKKIRKPRTIYSSFQLAALQRRFQQTQYLALPERAELAASLGVTQTQVKIWFQNRRSKFKKIGKHGEMMP---QASSPWD--PWFPSTYGPPP-LLHAPH---DVSATAAF
PmDlxA    ----VF-ECSYAYHQDKDDRQ-SGVRMNGKPKKIRKPRTIYSSFQLAALQRRFQQTQYLALPERAELAASLGLTQTQVKIWFQNRRSKFKKLGKNGEMAP---QASAPW-ELPWYRHS--PPS-LLALAQ---PECKWRHF
PmDlxB    ----------APYDADKDDSE-PEARLNGKPKKVRKPRTIYSSFQLAALQRRFERTQYLALPERAELAASLGLTQTQVKIWFQNRRSKFKRLVKSGELGSEHSPSPAPWDSSSWYAVSAA-VIY-----------------
MmDlx1    SGKAVFMEFGYPYV-EKSTVEGGEVRFNGKGKKIRKPRTIYSSLQLQALNRRFQQTQYLALPERAELAASLGLTQTQVKIWFQNKRSKFKKLMKQGGAALEGSPVPPGWNYVPWY---MQQPQ-LM---------------
HsDlx1    SGKAVFMEFGYPYV-EKSTVEGGEVRFNGKGKKIRKPRTIYCSLQLQALNRRFQQTQYLALPERAELAASLGLTQTQVKIWFQNKRSKFKKLMKQGGAALEGSPVPPGWNYIPWY---MQQPQ-LM---------------
HsDlx4    ASKAVFPDLAYPYT-EKPRPEPSERRPQAPAKKLRKPRTIYSSLQLQHLNQRFQHTQYLALPERAQLAAQLGLTQTQVKIWFQNKRSKYKKLLKQNSGGQEGDPLPSLWDYGNWY---LASPQ-MM---------------
MmDlx4    ASNVVFPDLAYSHP-EKLALVPSQQ--QSLTRKLRKPRTIYSSLQLQHLDQRFQHTQYLALPERAQLAAQLGLTQTQVKIWFQNKRSKYKKLLKQSSGEPEEDALPFIWGYDNWY---LALPQ-MM---------------
MmDlx6    SSKSAFMEFGSPYASQKTTIENGEIRFNGKGKKIRKPRTIYSSLQLQALNHRFQQTQYLALPERAELAASLGLTQTQVKIWFQNKRSKFKKLLKQGSNPHESDALPPVWDYMPWY---MQRPQ-MM---------------
HsDlx6    SSKSAFMEFGSPYASQKTTIENGEIRFNGKGKKIRKPRTIYSSLQLQALNHRFQQTQYLALPERAELAASLGLTQTQVKIWFQNKRSKFKKLLKQGSNPHESDALPPVWDYMPWY---MQRPQ-MM---------------
PfDlx     -SKSAFMEIQYPFHMEKTVSE-NELRVNGKGKKMRKPRTIYSSLQLQQLNRRFQRTQYLALPERAELAASLGLTQTQVKIWFQNRRSKYKKVLKQQQQHPHQPSSPPVWDYLPWY--TIQQPQ-LLT--------------
PmDlxD    H---HY----YPYL-EKTPH-NGELRFNGKGKKIRKPRTIYSSLQLQALNRRFQQTQYLALPERAELAASLGLTQTQVKIWFQNKRSKYKKLMKAGGAGLENNPQQAVWDYMPWY---MANQP-VM---------------
CeDll     -SKGFEYVYYYPPGPTKIV-EGCEAKYNVKGKKMRKPRTIYNSSQLQMLQKKFQKTQYLALPDRAALAHELGLSQTQVKIWFQNRRSKQKK-QKGGSSDHA-----ESVMYLY-Y---VYQNT-LY------------KYV
PlaDlxb   -SRAQFMHTSYPYPPAKEENE-PGMSSKDKRKKMRKPRTIYSSLQLQQLNKRFQRTQYLALPERAELAASLGVTQTQVKIWFQNKRSKFKKIVKHGGIPSQNQCQNPSVMYSTMYGGAASHMG-FVS--------------
NvDlx     ----PD-LAYHPLSHDKSS-C-DEPRLNGKGKKIRKPRTIYSSFQLRELNKRFIKTQYLALPERADLAAYLGLTQTQVKIWFQNRRSKFKKTLKVSEDNPVKEGQVEPWCFKP-Y-------S-AILR-------------
SkDll     -SKSAFMEIQYPFHMEKSASE-NELRVNGKGKKMRKPRTIYSSLQLQQLNRRFQRTQYLALPERAELAASLGLTQTQVKIWFQNRRSKCKKMLKQQQQQPHQTSSPPVWDYIPWY--ALQQSQ-LMS--------------
LgDll     ASKSAFMELQYPFPMDKSQVE-EQLRINGKGKKLRKPRTIYSSLQLQQLNRRFQRTQYLALPERAELAASLGLTQTQVKIWFQNKRSKYKKVMKQNPNGPPPPPPCS-WDYMSWY----QQ-Q-LLT--------------
BfDll     MSKSAFMEIPYPFSMDKSPTD---QRINGKGKKMRKPRTIYTSFQLQQLNRRFQRTQYLALPERAELAAQLGLTQTQVKIWFQNRRSKYKKLMKQGGAPPPGVSSPPVWDYMNWY----QQSQQLLT--------------
PduDlx1   VSKSAFMEIQYPFSMEKPSDE---LRVNGKGKKMRKPRTIYSSLQLQQLNKRFQRTQYLALPERAELAASLGLTQTQVKIWFQNRRSKYKKLMKQNPGIGGPGSPPVSWGYMSWY--AAQQHS-LLT--------------
DmDll     PGKSAFVELQYPFPPDKC--EDSGLRVNGKGKKMRKPRTIYSSLQLQQLNRRFQRTQYLALPERAELAASLGLTQTQVKIWFQNRRSKYKKMMKAAQGPGTNSPTQAPWDYVPWY---------LVTVWPAV---------
AgDll     PGKSAFVELQYPFPPDKL--EDTGLRVNGKGKKMRKPRTIYSSLQLQQLNRRFQRTQYLALPERAELAASLGLTQTQVKIWFQNRRSKYKKMMKAAQAPGVGGPSQAPWEYVPWY---------LLTVWPAV---------
ArDll     SGKSAFIELQYPFAPDKSG-GGGSLR-NGKGKKMRKPRTIYSSLQLQQLNRRFQRTQYLALPERAELAASLGLTQTQVKIWFQNRRSKYKKMMKAAQQGGGGGPPAPVWPYMPWY---------LLTVWPAV---------
TcDll     VSKSAFIELQYPFRPEKCL--SLE-RPGGKGKKMRKPRTIYSSLQLQQLNRRFQRTQYLALPERAELAASLGLTQTQVKIWFQNRRSKYKKMMKAAQVSGGNNRPKHDGR---TP---------LFNTEESD---------
DpuDll    ASKNAFLELQYPFSHDKDS-ESG-IRVNGKGKKMRKPRTIYSSLQLQQLNRRFQRTQYLALPERAELAASLGLTQTQVKIWFQNRRSKYKKMMKAAQQVGGGGSTGGSWDYMPWY---------LLT--------------
TadDll    ASKSAFVEVKTPYVSEKT--GNHKPR-SNKGKKNRKPRTIFTSAQLNELNNRFRLSHYLGLPERADLAASLGLTQTQVKIWFQNRRSKLKKSTGGRG------MQNQSWN---WYSLQASQHQ-LFPN-------------
CapDlxb   -KLSSFMDIPYSFQMDKTQ-DEGSLRMNGK-KKMRKPRTIYSSLQLQQLNRRFQRTQYLALPERAELAASLGLTQTQVKIWFQNKRSKYKKIMKQGQTPGGPPAANNAWDYSPWY---------IPS--------------
PlaDlxa   -SKSAFLDLQYPFGMSKQV-DEPTPKGSGK-KKIRKPRTIYSSLQLQQLNKIFQRTQYLSLPERAELAASLGLTQTQVKIWFQNKRSKFKKILKQQQPGGGPLGAGSSWDYMPWY----SQSS-LLT--------------
CapDlxa   -TKSAFLDLQQ--G-EKTN-DDPNVRIPNK-KKMRKPRTIYSSLQLQQLNRRFQRTQYLALPERAELAASLGLTQTQVKIWFQNKRSKCKKIMKQQGPGSTPASPPATW-NMYWY----HQSS-LLT--------------
CiDlla    PGNDVRPWVHYPGTTEKFNDTPSVQNGRVKGKKTRKPRTIYTSYQLQQLVRRFQRTQYLALPERAELAASLGVTQTQIKIWFQNRRSKYKKLLKQQLLHKHHGPESQGWD--AVD---MRRDG-ILQWGSP----------
SpDll     QHQAAMPYHHMSQTR-KEGSDTSIETKSLRGKKLRKPRTIYTSLQLQQLNQRFHQTQYLALPERAELAASLGLTQTQVKIWFQNRRSKYKKILKQQNQSNNNNTQHQQHDHKA-----SGPVG-GRRLG-P----------
CiDllb    SSNYSYLPYQTPXESPSSSTFNDTTEQTSKSKKKRNPRTVYSNYQLQELHMYFKKVQYLALPERARLAAALGLTQTQVKVWFQNRRSKIKKLLKSGVVQDLDEKQHCPSNFGVST---VDTPN-TFNSYKNN---------
BfMsx     ---SAFSK--PPSRPPRD-------RLLRKHKTNRKPRTPFTTQQLLALERKFRQKQYLSIAERAEFSASLNLTETQVKIWFQNRRAKAKRLQEAELEKLKMA-------------------FIHSS--------------
CiDllc    ----AA-SGYY----LHQD---SDVLGKGKKKKMRKPRTIYSSLQLQALNRRFQQTQYLALPERAELAATLGLTQTQVKIWFQNRRSKCKKLMKQGIHDKDNPPQPTSWTHPSWY----QQNLQHMENRGIR---------
HrDlxβ    SVTSQVSISSNKNNNLDNIQPENEHVGLVGKKKMRKPRTIYSSLQLQQLNRRFQRTQYLALPERAELAASLGLTQTQVKIWFQNRRSKVKKLIKQQCTGSSSN-------YLPWH-----ACNQLY---------------
HrDlxα    NHIQQHQQQLNNNNNSMQQ-QQQDGR-INSKKKMRKPRTIYSSLQLQQLNRRFQRTQYLALPERAELAASLGLTQTQVKIWFQNRRSKYKKINKQPGNQATSTCKSEFWDYPQWF-----NNNNNICNNNNS---------
```

Additional File 6. Alignment of homeodomain and other conserved regions of Dlx genes used for phylogenetic analysis.
