# Supplementary material for: Annelid Distal-less/Dlx duplications reveal varied post-duplication fates
Source: BMC Evol Biol. 2011 Aug 16;11:241. doi: 10.1186/1471-2148-11-241 (PMC3199776; doi:10.1186/1471-2148-11-241)
Supplement: Additional file 7 — Geneconv output. Tracts of Dlx sequences likely to be undergoing gene conversion as predicted by Geneconv. [file 1471-2148-11-241-S7.PDF]

Additional File 7. Tracts of Dlx sequences likely to be undergoing gene conversion as predicted by Geneconv.

Sequence	Tract length (bp)	P value (global)	Region of gene
CtDlxa;CtDlxb	150	0.0000	2nd exon, 5' homeodomain and part of intron
CtDlxa;CtDlxb	31	0.0000	3rd exon, 3' homeodomain
PlaDlxa;PlaDlxb	21	0.0000	2nd exon, 5' homeodomain
CtDlxa;CtDlxb	17	0.0000	Intron between exons 2 and 3
PlaDlxa;PlaDlxb	17	0.0000	3rd exon
CtDlxa;CtDlxb	15	0.0000	3rd exon, 3' homeodomain
PlaDlxa;PlaDlxb	15	0.0000	3rd exon, 3' homeodomain
PlaDlxa;PlaDlxb	14	0.0002	3rd exon, 3' homeodomain
PlaDlxa;PlaDlxb	13	0.0007	2nd exon, 5' homeodomain
PlaDlxa;PlaDlxb	11	0.0077	2nd exon, 5' homeodomain
PlaDlxa;PlaDlxb	11	0.0077	3rd exon, 3' homeodomain and 3 bp of intron
PlaDlxa;PlaDlxb	10	0.0264	2nd exon, 5' homeodomain
