# Supplementary material for: Annelid Distal-less/Dlx duplications reveal varied post-duplication fates
Source: BMC Evol Biol. 2011 Aug 16;11:241. doi: 10.1186/1471-2148-11-241 (PMC3199776; doi:10.1186/1471-2148-11-241)
Supplement: Additional file 8 — Hep-like domains. Alignment of N-terminal Hep-like domains of selected homebox and forkhead genes. [file 1471-2148-11-241-S8.PDF]

```
eh1             HRALPFSIDNILSLDFGRRKKVS
TN                 TPFSVKDILNLE
EH1                LAFSIDNILSPEFG
Octapeptide        SSFSIDGILG
Hep(A)             LKFSIDNILSAEF
Hep(B)             LKFSIDRILSAEF
HNF-3            FNHPFSINNLMS

HsNKX2.3        VTSTPFSVKDILNLEQQHQHFHG
DmScro          NHSTPFSVTDILSPIEESYRKLE
AgNK3           IVSTPFSINDILTRSRRVERHSS
HsNKX3.1           KPLTSFLIQDILRDGAPEEAETL
HsNKX3.2     NTLTSFSIQAILNKKEERGGLAA
HsLbx1             KPLTPFSIEDILNKPSVRRSYSL
AgTlx              NENLPFSISRLLGKSYDRDQKDK
NvNk1        PVVTSFSVKDILDPNKFTASTIR
OlGsh1              MPRSFLVDSLILREANEKGS
DrVent          MIPSKFSVEWLSQSFHDQEKCST
XlVent1b        MVQQGFSIDLILARSKEEAADGK
XlVent2         ~MTKAFSSVEWLAQSSRRSHREQ
TadDlx           PASKSAFVEVKQPSLYP
BfDlx        MSKSAFMEIPVQQTPVSMSMSM
PduDlx            VSKSAFMEIQQQQMNASMNPYA
DmDll       PGKSAFVELQQHAAAGYGGIRS
BfMsx        SNSAFSKPTTSAPSSASSPTS
```

**Additional File 8. - Alignment of N-terminal Hep-like domains of selected homebox and forkhead genes.**

Published consensus sequences are underlined; these include eh1, TN, EH1, octapeptide, Hep (A and B) and HNF-3. Genbank accession numbers of examples are as follows: HsNKX2.3 AAF44651, DmScro NP_001104400, AgNK3 XP_313453, HsNKX3.1 AAG39735, HsNKX3.2 NP_001180, HsLbx1 NP_006553, AgTlx XP_313455, NvNk1 AAP88429, OlGsh1 NM_001104833.1, DrVent NP_571775, XlVent1b Q9YH71, XlVent2 AAI57738, TadDlx ABC86114, BfDlx P53772, PduDlx CAJ38799, DmDll AAB24059, BfMsx CAA10201.
