# Supplementary material for: Annelid Distal-less/Dlx duplications reveal varied post-duplication fates
Source: BMC Evol Biol. 2011 Aug 16;11:241. doi: 10.1186/1471-2148-11-241 (PMC3199776; doi:10.1186/1471-2148-11-241)
Supplement: Additional file 9 — Sequences used in phylogenetic analyses. Accession numbers and database sources for sequences used in phylogenetic analyses. [file 1471-2148-11-241-S9.PDF]

**Additional File 9. Accession numbers (Genbank) and database sources (JGI) for sequences used in phylogenetic analyses.**

| Name | Species | Accession/scaffold details |
|---|---|---|
| AgDll | *Anopheles gambiae* | EAA03995.2 |
| ArDll | *Athalia rosae* | BAG06741.1 |
| BfDll | *Branchiostoma floridae* | Brafl1/scaffold_53:680733-686543 v1.0 |
| BfMsx | *Branchiostoma floridae* | CAA10201.1 |
| CeDll | *Caenorhabditis elegans* | NP_497904.1 |
| CiDlla | *Ciona intestinalis* | Cioin2/chr_07q:1163932-1166747 V2.0 |
| CiDllb | *Ciona intestinalis* | ciona4/Scaffold_10:13952-16749 V1.0 |
| CiDllc | *Ciona intestinalis* | Cioin2/chr_07q:4208077-4220106 V2.0 |
| DmDll | *Drosophila melanogaster* | AAB24059.1 |
| DpuDll | *Daphnia pulex* | Dappu1/scaffold_121:199759-203057 v 1.0 |
| HsDlx1 | *Homo sapiens* | AAH36189.2 |
| HsDlx2 | *Homo sapiens* | AAH32558.1 |
| HsDlx3 | *Homo sapiens* | AAH28970.1 |
| HsDlx4 | *Homo sapiens* | EAW94654.1 |
| HsDlx5 | *Homo sapiens* | AAP35549.1 |
| HsDlx6 | *Homo sapiens* | EAW76744.1 |
| LgDll | *Lottia gigantea* | Lotgi1/sca_12:2971496-2981701 |
| MmDlx1 | *Mus musculus* | NP_034183.1 |
| MmDlx2 | *Mus musculus* | NP_034184.1 |
| MmDlx3 | *Mus musculus* | NP_034185.1 |
| MmDlx4 | *Mus musculus* | NP_031893.1 |
| MmDlx5 | *Mus musculus* | NP_034186.2 |
| MmDlx6 | *Mus musculus* | NP_034187.1 |
| NaDll | *Neanthes arenaceodentata* | ACN66454.1 |
| NvDll | *Nematostella vectensis* | Nemve1/scaffold_20:1051430-1053020 v 1.0 |
| PduDlx1 | *Platynereis dumerilii* | CAJ38799.1 |
| PfDlx | *Ptychodera flava* | BAA89014.1 |
| PmDlxA | *Petromyzon marinus* | AAG41495.1 |
| PmDlxB | *Petromyzon marinus* | AAG41496.1 |
| PmDlxC | *Petromyzon marinus* | AAG41497.1 |
| PmDlxD | *Petromyzon marinus* | AAG41498.1 |
| SkDll | *Saccoglossus kowalevski* | AAP79300.1 |
| SpDll | *Strongylocentrotus purpuratus* | XP_782745.1 |
| TadDll | *Trichoplax adhaerens* | ABC86113.1 |
| TcDll | *Tribolium castaneum* | AF317551_1 |
